# Adaptive behaviors of *Drosophila* larvae on slippery surfaces

**DOI:** 10.1007/s10867-023-09626-2

**Published:** 2023-02-15

**Authors:** Li Guo, Yixuan Sun, Sijian Liu

**Affiliations:** Zhejiang Lab, Nanhu Headquarters, Kechuang Avenue, Zhongtai Sub-District, Yuhang District, Hangzhou City, Zhejiang Province 311121 People’s Republic of China

**Keywords:** *Drosophila*, Slipping, Locomotion, Adaptation, Paradigm

## Abstract

Friction is ubiquitous but an essential force for insects during locomotion. Insects use dedicated bio-mechanical systems such as adhesive pads to modulate the intensity of friction, providing a stable grip with touching substrates for locomotion. However, how to uncover behavioral adaptation and regulatory neural circuits of friction modification is still largely understood. In this study, we devised a novel behavior paradigm to investigate adaptive behavioral alternation of *Drosophila* larvae under low-friction surfaces. We found a tail looseness phenotype similar to slipping behavior in humans, as a primary indicator to assess the degree of slipping. We found a gradual reduction on slipping level in wild-type larvae after successive larval crawling, coupled with incremental tail contraction, displacement, and speed acceleration. Meanwhile, we also found a strong correlation between tail looseness index and length of contraction, suggesting that lengthening tail contraction may contribute to enlarging the contact area with the tube. Moreover, we found a delayed adaptation in *rut* mutant larvae, inferring that neural plasticity may participate in slipping adaptation. In conclusion, our paradigm can be easily and reliably replicated, providing a feasible pathway to uncover the behavioral principle and neural mechanism of acclimation of *Drosophila* larvae to low-friction conditions.

## Introduction

Insects have a highly adaptive motor control capability, which quickly acclimates to challenging situations. Insects routinely generate rhythmic and stereotyped motor behaviors [1–3]. However, these patterns can be reshaped by central pattern generators (CPGs) to accommodate the new environment [4–6]. Exposed to nociceptive stimuli such as heat or challenging tasks such as crossing large gaps, insects’ CPGs will be upgraded to coordinate with sensory feedback [7, 8]. For example, *Drosophila* larvae can evade direct heavy winds by altering the probability, direction, and size of turns [9]. In another case, *Drosophila* manages to cross large gaps by making small, continuous modifications to the bending angles of the body and frontal legs [7].

Static friction with various contact substrates is an indispensable force for insect locomotion such as climbing, gripping, and walking [10–12]. Due to unpredictable environments and diverse touching textures, insects must cope with ever-changing friction to maintain stability in their gait or posture. Interestingly, insects have evolved a superb system of friction regulation, such as chemical glues and releasable pads, which allow them to instantly modify static friction to achieve body balance or solid grip [13, 14]. Although many studies have been conducted to provide detailed ethological and morphological descriptions and explanations of friction regulation, little is known about how neural circuits integrate multiple sensory modal inputs to generate coordinated behavior sequences that fulfill rapid adhesion regulation. A critical limitation in dealing with this problem arises from the lack of a suitable paradigm to create a reliable low- or high-friction condition to quantify fine motor adjustment and monitor neural activity of behaving insects. Previous paradigms had been developed to investigate segment kinematics of forward crawling under “no friction” rather than low-friction conditions by floating larvae in liquid. However, this paradigm prevented larvae from performing effective behavior alternations to achieve sufficient friction [15].

*Drosophila melanogaster* larva, a representative insect with a miniature nervous system and stereotyped behaviors, is an ideal animal to reveal the neural basis for the emergence of new motor patterns during friction adaptation. Here, we have developed a novel behavior paradigm to induce *Drosophila* larvae to exhibit slipping-like behaviors under low-friction conditions. We discovered a novel phenotype, tail looseness, to evaluate the degree of slipping. We found that larvae gradually reduced slipping-like behavior after initial trials, coupled with increased tail contraction, displacement, and speed. In addition, we observed that lengthening tail contraction is related to larger contact areas, which probably generated more static friction to anchor posterior segments to promote slipping adaptation. Our paradigm can be reliably replicated as the arena can be effortlessly constructed and lubricant reagents are widely used in the laboratory. Hopefully, the paradigm may pave the way for mapping sensorimotor circuits in *Drosophila* larvae that generate adaptation under slippery conditions.

## Materials and methods

We have found two methods to efficiently elicit larvae to exhibit slipping behaviors. The first verified method was performed on a Petri dish with a thin coating of 16 g/L hydroxyethyl starch, glycerin, or mineral oil. Typical features of larval slipping on the dish substrate include swing-tails, unbalanced body, and ineffectiveness in moving forward. The second method (see more details in the section), slightly modified from the first to deliberately restrain tail bending and body roll, was performed in an FEP tube filled with 16 g/L hydroxyethyl starch or mineral oil. Here, we detail the procedure on how to induce larvae slipping in the tube, and reveal fined behavioral alternations undertaken by larvae to achieve slipping adaptation.

### Slipping-inducing setup

**Fig. 1:**
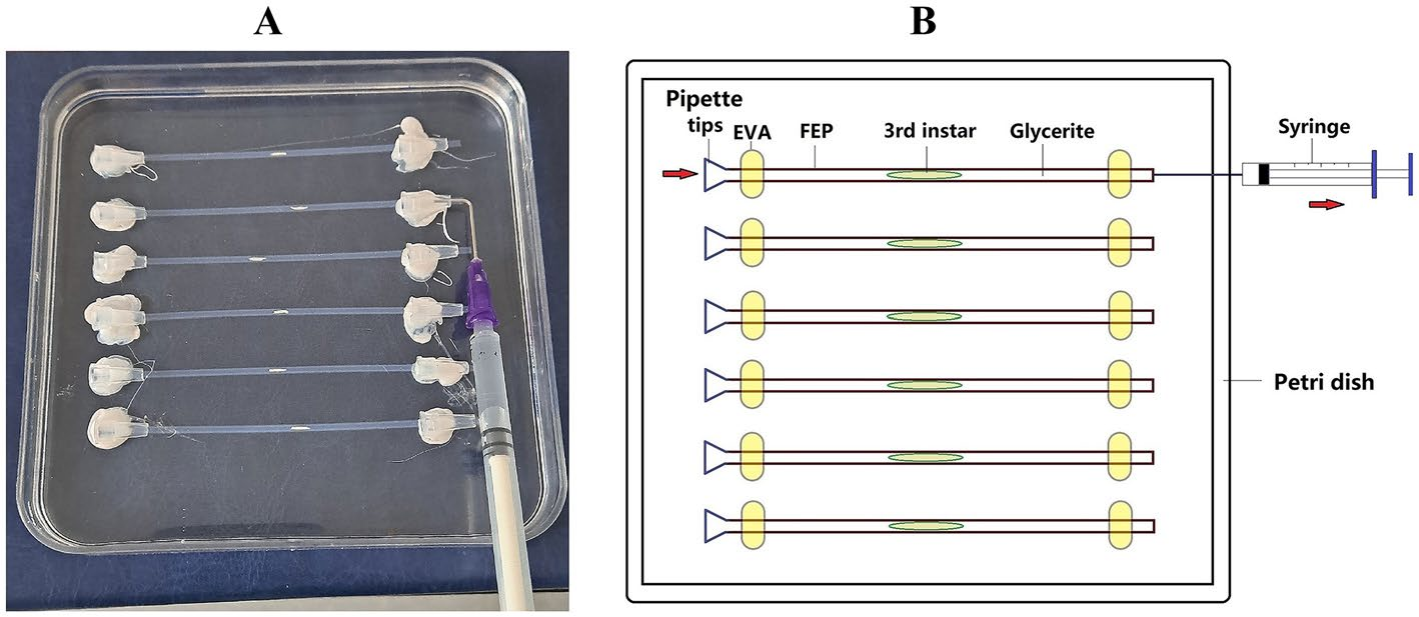
Schematics and picture of the arena for inducing larvae slipping. **A** Picture of the slipping-inducing arena. Prior to the behavior test, the FEP tube was filled with lubricant such as 16 g/L hydroxyethyl starch. 3rd instar was dragged into the middle of the tube from pipette tips by a syringe. **B** Schematics of the slipping-inducing arena. The setup consisted of FEP tubes (diameter*length, 0.8 cm*10 cm) attached to the Petri dish (length*length, 13 cm*13 cm) by melted EVA (yellow)

The slipping-inducing arena was designed to observe and record the locomotion of 3rd instar crawling in the man-made low-friction conditions. The arena was constructed on a plastic Petri dish (length*length, 13 cm*13 cm) with six parallel lined fluorinated ethylene-propylene (FEP) tubes (internal diameter = 800 μm) attached to its bottom surface (Fig. 1A). FEP tubes were adhered to the dish with HMA (hot melt adhesive). 3rd instar was dragged in or out of the tube via pipette tips by a 1.5-mL syringe (Fig. 1B).

### Procedure of behavioral assays

We had identified several ideal reagents such as 10% (w/v) sodium dodecyl sulfate (SDS), mineral oil, and 16 g/L hydroxyethyl starch as they had verified friction-reducing effect as well as biological safety. At the same time, we selected 3rd *w*^1118^ instars as its approximate FEP tube diameter, which preserved sufficient room for posture adjustment to achieve slipping adaptation.

The low-friction conditions was mimicked at the slipping-inducing arena filled with 16 g/L hydroxyethyl starch. After rinsing with tap water for 5 min, the arena was poured with 16 g/L hydroxyethyl starch to submerge the FEP tube. Air bubbles stuck in the tube should be completely removed by flowing lubricant with the help of a syringe.

*Drosophila melanogaster* was raised under conditions of 25 ℃, 60% humidity, and a 12:12-h light–dark cycle (light was set at 8:00 to 20:00). Foraging 3rd instar was separated from the bottle by 1 mL of tap water and then collected by a paintbrush. After being rinsed in water for 60 s, the larvae were transferred to tissue paper to soak up excess water, and then gently dragged into the FEP tube by syringe.

The imaging equipment for recording larval locomotion was a stereomicroscope, camera Motic (500 million pixels). The camera (Motic, 500 million pixels) was connected to a computer via a data cable (USB 2.0) for harvesting high-quality videos for behavior analysis. The field of observation and video recording of behaving larvae was maintained at the center by adjusting the camera and microscope (Motic). Meanwhile, high-quality larval locomotion videos were obtained by modifying camera settings via Swift EasyView software. Once the above preparation was completed, video recording was immediately started and the larvae were dragged into the tube. The videos were recorded at a frame rate of 7 Hz and lasted 7–10 min.

### Quantitative behavioral analysis of larval locomotion

After recording larval locomotion in the low-friction arena, the newly captured mp4-format videos were converted to avi (mjepg) by Apowersoft software, and again converted to tiff-format image sequence by software ImageJ. To quantify larval locomotion such as contraction, displacement, and speed, we selected posterior spiracles on the tail as distinguishable reference points to mark the larval position. After marking the posterior spiracles as region of interest (ROI) frame by frame using ImageJ, we measured the position (*x*/*y* coordinates) of each larva.

**Fig. 2:**
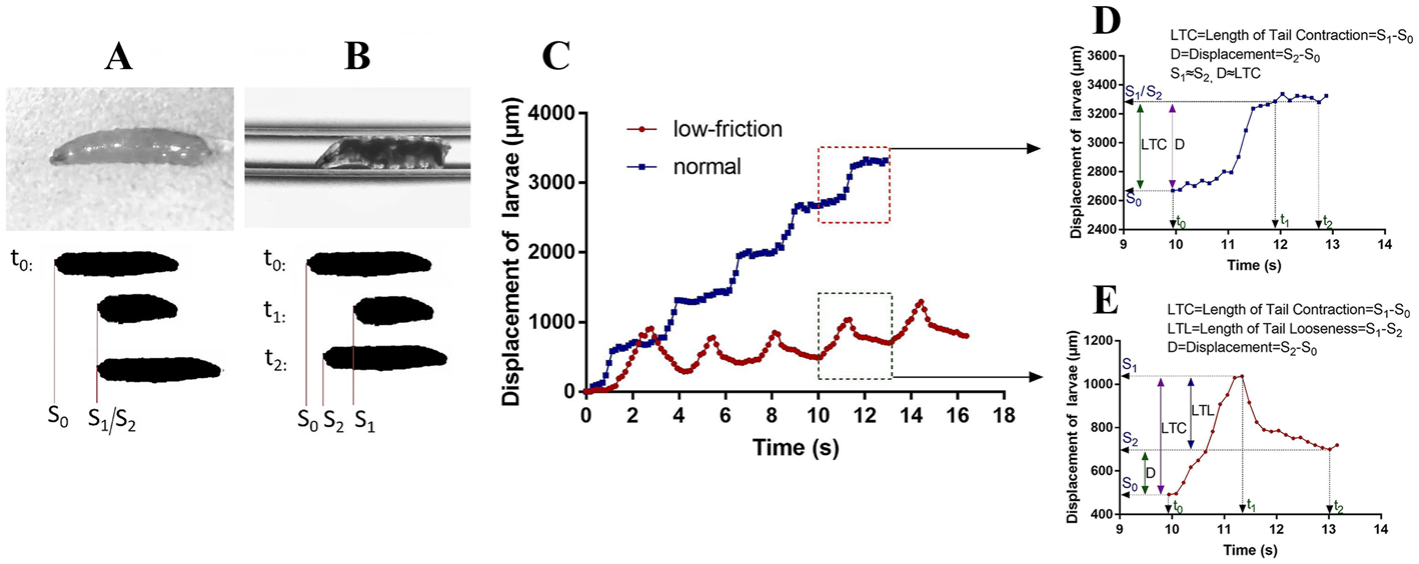
*Drosophila* larvae displayed typical tail looseness under low-friction surfaces. **A**, **B** Screenshots of larvae crawling on the rough Petri dish (upper left panel) and lubricant-filled FEP tube (upper right panel). Diagram depicted the tail position of larvae in a trial under normal (lower left panel) and low-friction (lower right panel) conditions. At *t*_0_, larvae were preparing for a stride wave and the tail position was marked *S*_0_. At *t*_1_, larvae had just accomplished tail contraction and the tail position was marked *S*_1_. At *t*_2_, larvae finished tail looseness and the tail position was marked *S*_2_. **C** Displacement of larvae in 5 consecutive trials under normal (blue) and low-friction (red) conditions. **D** Displacement of larvae at 9.94–13.02 s (extracted from **C**, normal) under normal conditions. LTC, length of tail contraction = *S*_1_ − *S*_0_; *D*, displacement = *S*_2_ − *S*_0_. Since larvae did not exhibit tail looseness under rough surfaces, *S*_1_ ≈ *S*_2_ and *D* ≈ LTC. **E** Displacement of larvae at 9.94–13.02 s (extracted from **C**, low-friction) under low-friction conditions. LTC, tail contraction = *S*_1_ − *S*_0_; LTL, length of tail looseness = S_1_ − *S*_2_; *D* = displacement = *S*_2_ − *S*_0_

Crawling on rough surfaces (Fig. 2A, upper panel), *Drosophila* larvae displayed periodic forward strides propelled by tight tail grip (Fig. 2A, lower panel; Fig. 2C). However, confined to the FEP tube filled with lubricant (Fig. 2B, upper panel), larvae cannot firmly anchor their abdominal tentacles and tail due to the low-friction surfaces, resulting in tail loosening (Fig. 2B, lower panel; Fig. 2C) after completion of their contraction for peristaltic wave. To assess the intensity of slipping-like behavior and changes in locomotion patterns, we introduced the following six metrics: displacement, speed, duration, length of tail looseness, tail contraction, tail looseness index, in a single trial.

The length of tail contraction (LTC, *S*_1_ − *S*_0_, Fig. 2E) was measured by the distance between the initial position (*S*_0_, Fig. 2E) at the start of the trial and the last position (*S*_1_, Fig. 2E) of spiracles when the contraction ended. Tail looseness (TL, *S*_1_ − *S*_2_, Fig. 2E) was calculated by the length of tail withdrawal during peristaltic wave propagation following tail contraction in an individual trial. The tail looseness index (TLI, (*S*_1_ − *S*_2_)/(*S*_1_ − *S*_0_), Fig. 2E) was calculated as the ratio of length of tail looseness (*S*_1_ − *S*_2_, Fig. 2E) to contraction (*S*_1_ − *S*_0_, Fig. 2E). The displacement (*S*_2_ − *S*_0_, Fig. 2E) in a single trial was measured by the distance between the initial position (*S*_0_, Fig. 2E) at the start of the trial and the last position (*S*_2_, Fig. 2E) of spiracles at the end of a trial. The speed of a single trial (SST, (*S*_2_ − *S*_0_)/(*t*_2_ − *t*_0_), Fig. 2E) was measured by tail displacement (*S*_2_ − *S*_0_, Fig. 2E) divided by a trial duration (*t*_2_ − *t*_0_, Fig. 2E).

## Results

### Modification of motor patterns in larvae acclimation to the low-friction conditions

**Fig. 3:**
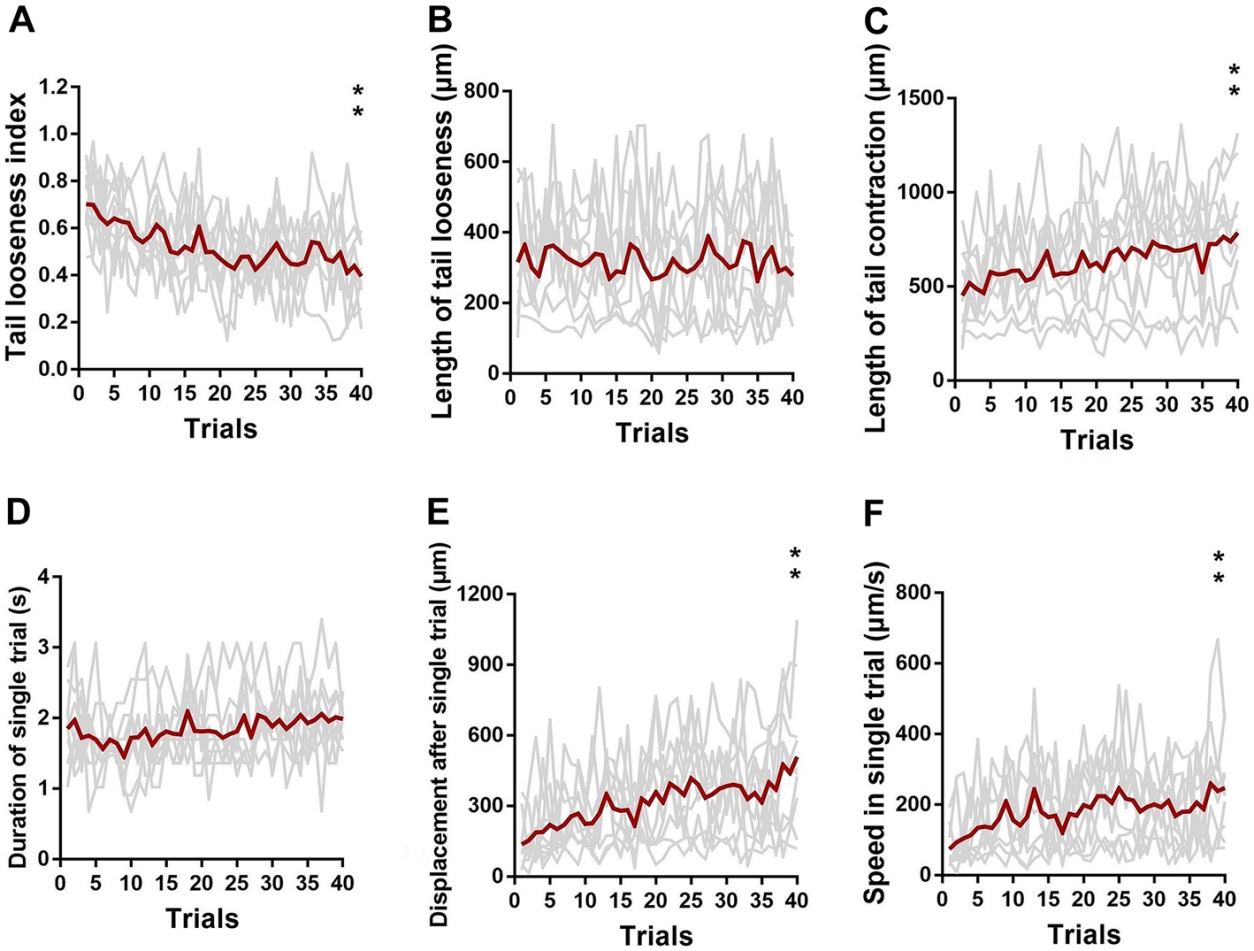
Alternation of locomotion patterns during larval acclimation to slippery conditions. **A**–**F** Tail looseness index, length of tail looseness, length of tail contraction, duration of single trial, displacement after single trial, and speed in single trial in 40 trials. Statistical significance was ascertained by the Wilcoxon matched-paired signed rank test, *n* = 10, **P ≦ 0.01

To quantify the degree of slipping phenotype and modification of peristalsis pattern in each trial, we devised six behavioral metrics (more details in Section 2) based on the length and duration of tail contraction, looseness, and displacement. Since tail looseness was the primary phenotype of slipping-like behavior, we used the tail looseness index (TLI) to weigh the slipping level. The TLI of wild-type larvae decreased inconsistently from 0.7028 ± 0.0428 (1st trial) to 0.3956 ± 0.0411 (40th trial) (Fig. 3A). However, the length of tail looseness in wild-type larvae was generally unaltered throughout the trials (Fig. 3B). The length of tail contraction and the duration of single stride wave were the critical indicators for the peristalsis pattern. The tail contraction of wild-type larvae was substantially prolonged from 452.24 ± 73.34 µm (1st trial) to 785.79 ± 110.00 µm (40th trial) (Fig. 3C). Meanwhile, their single wave duration fluctuated persistently around 1.83 s (Fig. 3D). Displacement and speed in a single trial directly indicated an improvement in slipping phenotype. We observed an increase in displacement from 136.83 ± 34.66 µm (1st trial) to 508.26 ± 100.75 µm (40th trial) and acceleration from 73.63 ± 17.99 µm/s (1st trial) to 246.90 ± 43.18 µm/s (40th trial) (Fig. 3E, F).

### Slipping level had strong correlations with tail contraction, displacement, and speed

**Fig. 4:**
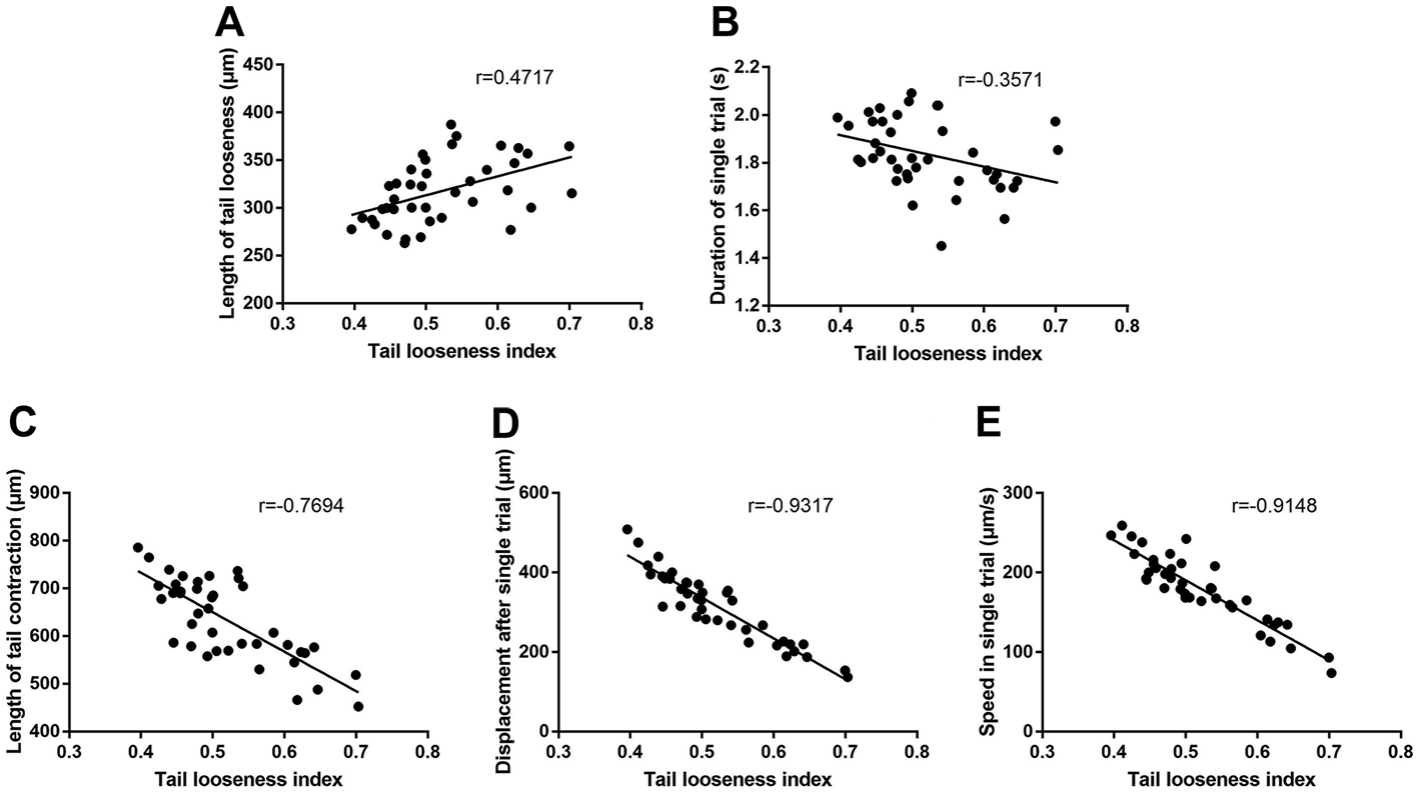
Correlation analysis on *w*^*1118*^ larvae between the tail looseness index and five other motor parameters. **A**–**E** Pearson correlation analysis between the tail looseness index and length of tail looseness, duration of single trial, length of tail contraction, displacement after single trial, and speed in single trial. Correlation analysis was conducted by treating TLI as an independent variable (*x*) and five other parameters as dependent variables (*y*)

We found that some behavior parameters shared similar or opposite trends, such as TLI vs LTC. To quantify their correlation, we conducted a correlation analysis between tail looseness index and the other 5 parameters. We found strong correlations between tail looseness index and length of tail contraction (*r* = ** −**0.7694, *P* < 0.0001; Fig. 4C), tail looseness index and displacement after single trial (*r* =  −0.9317, *P* < 0.0001; Fig. 4D), and tail looseness index and speed in single trial (*r* =  −0.9148, *P* < 0.0001; Fig. 4E). We also found moderate correlations between tail looseness index and tail looseness (*r* = 0.4717, *P* = 0.0021; Fig. 4A), and tail looseness index and duration of single trial (*r* =  −0.3571, *P* = 0.0237; Fig. 4B).

### Impaired synapse plasticity attenuated slipping adaptation but largely unchanged motor patterns

**Fig. 5:**
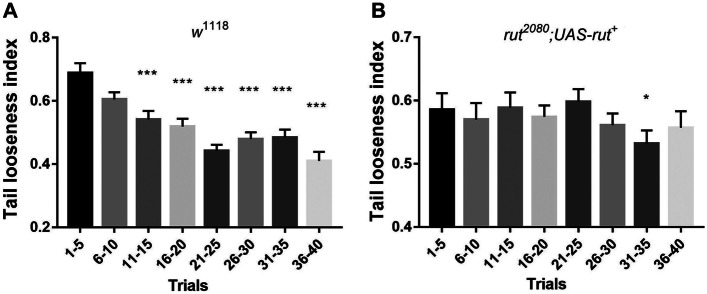
Dysfunction of *rut*-encoded neural plasticity delayed adaptation to slippery conditions. **A**, **B** Histograms illustrated tail looseness index of wild-type (*w*^1118^) and *rut* mutant (*rut*^*2080*^; *UAS-rut*.^+^), which were presented as mean values with 5-trial intervals: trials 1–5, 6–10, 11–15, 16–20, 21–25, 26–30, 31–35, 36–40. Statistical significance was determined by the Mann–Whitney test, *n* = 50, *N* = 10, *P ≦ 0.05, ***P ≦ 0.001

We observed larvae continuously adjusting their posture and contraction length in the low-friction conditions, suggesting that neural plasticity may be involved in the slipping acclimation. To test this hypothesis, we used larvae inherited with mutant *rutabaga* (*rut*), a key gene encoding calcium-sensitive-dependent adenylate cyclase, to examine impairment in slipping adaptation. We found that an initial significant drop in the tail looseness index in wild-type larvae occurred in trials 11–15 (Fig. 5A), while the initial decline in *rut* mutant larvae was delayed in trials 31–35 (Fig. 5B). These results suggested that dysfunction of neural plasticity may hinder the learning ability to gain acclimation to the low-friction surfaces, resulting in prolonged slipping adaptation.

**Fig. 6:**
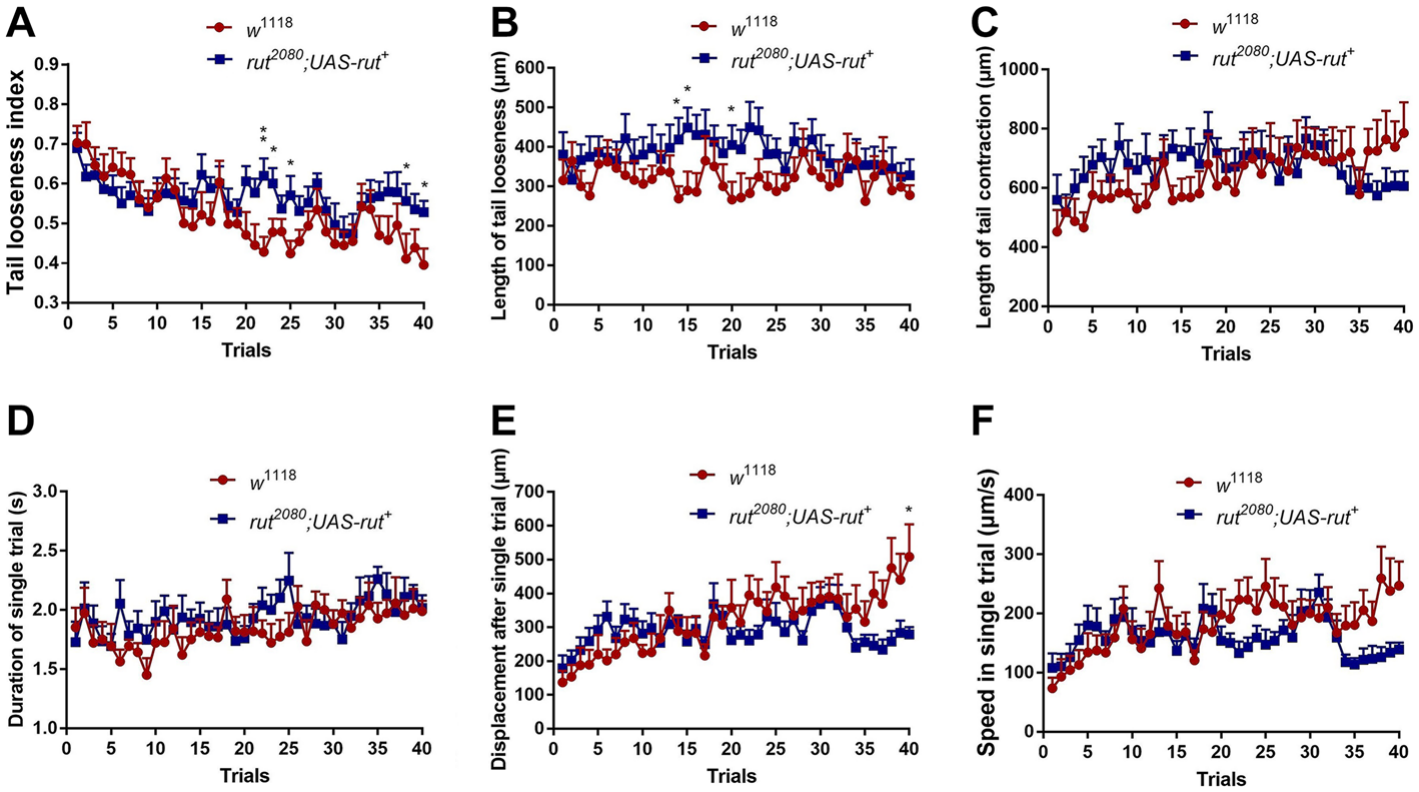
Dysfunction of neural plasticity attenuated slipping adaptation but largely unchanged motor patterns. **A**–**F** Tail looseness index, length of tail looseness, length of tail contraction, duration of single stride, displacement, and speed of wild-type (*w*^1118^) and *rut* mutant (*rut*^*2080*^; *UAS − rut*^+^) in 40 trials. Statistical significance was determined by the Mann–Whitney test, *n* (*w*^1118^) = 10, *n* (*rut* mutant) = 10, *P ≦ 0.05, ***P ≦ 0.001

In addition, we found that *rut* mutant larvae (Fig. 6A, blue) had a higher level of slipping (tail looseness index) than wild-type larvae (Fig. 6A, red) in the 22nd, 23rd, 25th, 38th, and 40th trials. However, *rut* mutant larvae generally maintained their motor patterns through 40 trials. However, compared to wild-type larvae, *rut* mutant larvae showed slightly larger tail looseness in the 14th, 15th, and 20th trials, and reduced displacement in the 40th trial.

## Discussion

### Abnormal locomotion and adaptation under low-friction surfaces

Foraging on rough solid surfaces, *Drosophila* larvae exhibited typical rhythmic crawling (Fig. 2A, C), noted by periodical propagation of peristaltic wave propelled by tail contraction. However, such a regular locomotion pattern can be temporarily reshaped by external perturbations such as heavy wind or appetizing odors [9, 16–18]. Under our paradigm, larvae were exposed to low-friction contact surfaces. We found larvae exhibiting a novel phenotype named tail looseness, a major abnormal behavior phenotype that tail slid toward the posterior after finishing contraction (Fig. 2B, C), similar to pedestrian slipping on snow-covered roads. Therefore, we defined this phenotype as a core feature of larval slipping-like behavior under low-friction conditions. Crawling within the low-friction FEP tube, larvae exhibited unique locomotion and alternating contraction-looseness patterns (Fig. 2C). After several trials, tail contraction gradually lengthened while looseness remained largely unchanged (Fig. 3B, C). Considering that TLI had a strong correlation with LTC and our empirical observation on larvae posture modification, we hypothesized that increasing contraction length contributed to a thicker diameter of A1–8 segments and greater pressure force on the tube, resulting in a larger contact area and static friction. Consequently, the larvae obtained sufficient friction to anchor the posterior segments (A6–8) on the tube, further promoting the displacement and speed of the single trial.

Static friction results from the interconnection of irregularities on touching surfaces [19], providing insects with a solid attachment for locomotion such as walking and crawling [20–22]. For instance, cockroaches have special attachment systems for self-stabilization. The adhesive pads on the distal foot covered with hairy ultra-structure produce tight adhesion to resist pulling forces [13]. Since larvae spend most of their time foraging in the culture bottle, they are likely to encounter slippery conditions similar to those in the lubricated tube, such as climbing on the wet surfaces of the culture bottle. Low-friction adaptation may facilitate larvae crawling safely and molting on the slippery bottle surfaces.

### Neural plasticity and some potential neural circuits promote slipping adaptation

We found an increase in tail contraction length but no significant change in stride wave duration in 40 trials (Fig. 3C, D), suggesting that interneurons controlling A1–8 segmental muscle contraction and relaxation maybe implicated in slipping adaptation. Since central pattern generators (CPGs) are widely thought to modulate rhythmic motor patterns, we proposed that these neurons may be components related to CPGs. To reveal the neural components involved in the slipping adaptation, a feasible solution is to combine loss-of-function (on neural transmission or plasticity-related gene expression) and behavioral testing to screen specific premotor or some CPG-related neurons.

Compared to wild-type larvae, *rut* mutants exhibited a slower and inconsistent tendency to decline on slipping level (Figs. 5B and 6A), indicating that *rut* gene was required for low-friction accommodation. We deemed such slippery acclimation as a special type of motor learning through progressive adjustment of posture and increment on length of contraction to enhance contact area and pressure force to generate sufficient static friction. Previous studies have found the essential role of neural plasticity in motor learning. For instance, training mice on rotor rods induced plasticity of neurotransmitter including amplified frequency and amplitude of AMPA-mediated EPSC and decreased presynaptic GABA release in M1 cortical neurons in layer II/III [23]. Learning novel motor tasks triggered changes in cAMP-mediated signals such as the cAMP/PKA/DARPP-32 pathway in the primary motor cortex and the cAMP-EPAC-PKCε-RIM1α pathway in the cerebellum, suggesting that cAMP played a critical role in the acquisition of new motor skills. Furthermore, upregulating cAMP expression levels in mice significantly alleviated motor learning deficits caused by loss of neurofibromin 1 (NF1) in D2R-MSN [24, 25]. Given that the *rut* gene encodes the calmodulin-activated adenylyl cyclase to synthetize cAMP and expresses pan-neurally like the mushroom and ventral nerve cord (VNC), it is imperative to use multiple genetic manipulations such as RNAi to dissect specific regions and components of circuits that modulate the reinforcement of specific posture and optimal locomotion pattern during the slipping adaptation. However, we found an intriguing rebound in the tail looseness index in trials 36–40 (Figs. 5B and 6A). One possible explanation is that *rut* mutant larvae cannot synthetize cAMP, resulting in inconsistent motor-related plasticity. In addition, the shortage of energy supply could also be an important factor as larvae spend more than 30 trials in the tube, resulting in a less stable tail anchor to the FEP tube and then larger tail looseness.

### Merits and some limitations of slipping-inducing paradigm

The slipping-inducing arena can be easily constructed and is user-friendly. The arena is assembled by inexpensive materials such as Petri dishes, FEP tubes, and reagents such as hydroxyethyl starch (Fig. 1). Moreover, the procedure for eliciting slipping phenotype and behavioral analysis is easy to perform, making our paradigm reliable to replicate. However, our paradigm still faces two major challenges to overcome. One problem is the lack of full perspective caused by the fixed camera setup, significantly hampering the capture of subtle locomotion such as alternation of touching area and posture adjustment. A practical solution is to set up three or more cameras at different angles to simultaneously capture 3D larval locomotion. Another solution to extend perspective is to rotate the tube steadily while keeping the camera still, which may help to obtain optimal observation of fine locomotion alternation during peristaltic waves.

Another challenge is precisely measuring static friction and effective attachment area during larvae slipping adaptation. Crawling in the tube filled with soliquid (16 g/L hydroxyethyl starch), the larvae must constantly modify posture to gain more contact area with the tube to generate sufficient static friction to anchor the body. Static friction results from the interaction between different larvae segments (such as anal pads from A8, tentacle bands from A1 to 8 segments, mouth hook) and the lubricated tube. However, currently no accessible methods have been published to accurately detect weak static friction and diminutive touching area of behaving insect larvae in liquid. In addition, *Drosophila* larvae have transparent skin, segmental tentacle bands, and spiny anal pads, making it difficult to distinguish the actual larvae boundary from the FEP tube and further accurately measure the contact area. Previous studies had utilized 2D-force transducers to capture touching area by coaxial illumination and monitor the faint static friction of *G*. *viridula* adhesive pads [26]. However, the main technical difficulty at present is the lack of a highly sensitive transducer and suitable microscope to capture such faint area-friction signals of fast crawling larvae. However, we are optimistic that more advanced solutions will emerge to precisely measure and calculate these parameters, helping to reveal the optimal body posture and locomotion pattern for slipping adaptation.

## Conclusion

We devised a novel paradigm by exposing *Drosophila* larvae to the lubricant-filled FEP tube. Under these low-friction conditions, larvae exhibited a unique phenotype called tail looseness similar to slipping-like behaviors in humans. To quantify slipping level and motor pattern alternation during crawling, we designed six behavioral metrics. Among these parameters, tail looseness index is a primary indicator to weigh the alleviation of slipping level. We found a gradual decline in the tail looseness index after successive trials, indicating that larvae gained acclimation to low-friction conditions. Meanwhile, we also found significant modification of motor patterns especially length of tail contraction, displacement, and speed. Interestingly, we also found a strong correlation between the tail looseness index and length of contraction, suggesting that lengthening tail contraction may contribute to enlarging the contact area with the FEP tube. To test the role of neural plasticity in low-friction adaptation, we compared *rut* mutant with wild-type larvae and found a delayed adaptation in *rut* mutant larvae, inferring that neural plasticity may participate in slipping adaptation. In conclusion, our paradigm provides a feasible solution to uncover the hidden behavioral principle and neural mechanism of larval acclimation to low-friction conditions.

## Data Availability

The authors confirm that all behavioral data to support the findings of our study are available in the paper.
